# Measuring Information Coupling between the Solar Wind and the Magnetosphere–Ionosphere System

**DOI:** 10.3390/e22030276

**Published:** 2020-02-28

**Authors:** Mirko Stumpo, Giuseppe Consolini, Tommaso Alberti, Virgilio Quattrociocchi

**Affiliations:** 1 Department of Physics, University of Rome Tor Vergata, Via della Ricerca Scientifica 1, 00133 Roma, Italy; mirko.stumpo@inaf.it; 2 INAF-Istituto di Astrofisica e Planetologia Spaziali, via del Fosso del Cavaliere 100, 00133 Roma, Italy; giuseppe.consolini@inaf.it (G.C.); virgilio.quattrociocchi@inaf.it (V.Q.); 3 Dipartimento Scienze Fisiche e Chimiche, Università degli Studi dell’Aquila, 67100 L’Aquila, Italy

**Keywords:** information theory, time series analysis, solar wind-magnetosphere–ionosphere system, space weather

## Abstract

The interaction between the solar wind and the Earth’s magnetosphere–ionosphere system is very complex, being essentially the result of the interplay between an external driver, the solar wind, and internal processes to the magnetosphere–ionosphere system. In this framework, modelling the Earth’s magnetosphere–ionosphere response to the changes of the solar wind conditions requires a correct identification of the causality relations between the different parameters/quantities used to monitor this coupling. Nowadays, in the framework of complex dynamical systems, both linear statistical tools and Granger causality models drastically fail to detect causal relationships between time series. Conversely, information theory-based concepts can provide powerful model-free statistical quantities capable of disentangling the complex nature of the causal relationships. In this work, we discuss how to deal with the problem of measuring causal information in the solar wind–magnetosphere–ionosphere system. We show that a time delay of about 30–60 min is found between solar wind and magnetospheric and ionospheric overall dynamics as monitored by geomagnetic indices, with a great information transfer observed between the *z* component of the interplanetary magnetic field and geomagnetic indices, while a lower transfer is found when other solar wind parameters are considered. This suggests that the best candidate for modelling the geomagnetic response to solar wind changes is the interplanetary magnetic field component $B_{z}$. A discussion of the relevance of our results in the framework of Space Weather is also provided.

## 1. Introduction

The response of the Earth’s magnetosphere–ionosphere system to the changes of solar wind and interplanetary medium conditions is the central issue of the studies in the framework of Space Weather. In the past decades a large amount of work has been done to provide new insights within the Space Weather discipline, e.g., the investigation, characterization and forecasting of physical processes operating in the near-Earth environment and their relation with the solar activity [1]. These include large-scale induced currents [2,3], geomagnetic storms and substorms [4], solar energetic particle events [5,6,7], and so on [8]. All these phenomena have a common driver which is the solar activity evolving on both temporal and spatial scales across the heliosphere, reaching the Earth’s boundary regions and interacting with the near-Earth electromagnetic environment. However, both the magnetosphere and the ionosphere do not passively respond to the solar wind variability but evidence a wide range of processes which are characterized by nonlinear and multiscale features [9,10]. Indeed, the magnetosphere–ionosphere system is characterized by both externally-driven and purely-internal processes (eventually triggered by interplanetary conditions changes), which are generally characterised by different spatial and temporal scales: while coherent intermittent internal processes occur on short timescales (τ< 100–200 min), the directly-driven processes from the solar wind occur on longer timescales [11,12]. In other words, the magnetosphere–ionosphere system displays a very complex dynamics, showing features that are due to the interplay of the external (interplanetary) driving and internal processes. The inherent complexity of the magnetosphere–ionosphere system dynamics manifests in scale-invariant and near-criticality features [13,14] that are not directly connected to the external driving. This suggests that the whole information content of the solar wind variability is not sufficient for a correct characterization and forecasting of the magnetosphere–ionosphere system; this aspect is clearly evidenced by the difficulty of reproducing the short timescale dynamics of geomagnetic indices only using solar wind plasma parameters [15].

Before the development of the Space Weather field almost all concepts from information theory were largely proposed, currently employed in several fields (e.g., [11,16,17,18,19]). These concepts mostly deal with the characterization of the behaviour of complex systems, e.g., physical systems consisting of many interacting components whose collective behavior is not the simple extrapolation of single-component one, with the main goal of understanding interactions among system components (e.g., [16,19]). Indeed, interacting systems and sub-systems can mutually exchange information, influence each other, and causally influence another one [19], thus proper measures of the information flow and of causality relationships were introduced (e.g., [20,21]). Moreover, complexity observed in such systems is not only related to their multi-component nature but also stems on their multiscale nature, in terms of both spatial and temporal scales, as well as on their nonlinear character. Thus, correctly investigating the multiscale nature of dynamical systems, properly identifying the main physical processes, and detecting cross-scale causal interactions (e.g., identify input and output system variables) are currently a challenge for dynamical system theory and play a crucial role for the Space Weather framework (e.g., [11,15,21]).

Recently, new approaches based on information theory concepts were proposed and successfully applied to unveil some features of the complex dynamics of the Earth’s magnetosphere–ionosphere system in response to solar wind changes [11,17,22,23,24,25]. These methods have been shown to be very successful in untangling some useful relations between different quantities. Motivated by these very promising results, in this work we present an application of these novel information theory approaches for understanding and characterizing the magnetosphere–ionosphere system response to interplanetary and solar wind changes. In particular, we present an application of the *transfer entropy* to the investigation of the relevant solar wind parameters affecting the multiscale fluctuations observed in the geomagnetic activity as monitored by SYM-H and AE indices. A time delay from 30 min up to ∼60 min is found between solar wind and magnetospheric overall dynamics as monitored by geomagnetic indices, in agreement with the typical response time of the Earth’s magnetosphere to the solar wind changes (e.g., [11]). Moreover, a great information transfer can be observed between the *z* component of the interplanetary magnetic field and geomagnetic indices, while a lower transfer is found when the Perreault-Akasofu coupling function, ε, is considered. This suggests that the best candidate for modelling the geomagnetic response to solar wind changes is the interplanetary magnetic field component $B_{z}$.

## 2. Data

For our analysis we use solar wind parameters obtained from spacecraft located at the L1 Lagrangian point (∼220 $R_{E}$ from the Earth) and shifted to the nose of the bow shock (∼14 $R_{E}$ from the Earth) as well as geomagnetic indices derived from ground-based observatories. Specifically, from the OMNI HR1 database, freely retrievable from the Space Physics Data Facility (SPDF) Coordinated Data Analysis Web (CDAWeb) interface at https://cdaweb.gsfc.nasa.gov/index.html/, we select 5-minute time resolution data related to the solar wind bulk speed and to the interplanetary magnetic field components (in the GSM reference frame). By means of these parameters we evaluate the Perreault-Akasofu coupling function [26], well-known as ε parameter, which is defined as

(1) $$\varepsilon =C_{0}\;v\;B^{2}\;\sin^{4}\left(\frac{\theta_{c}}{2}\right),$$

being $C_{0}=\frac{4\pi }{\mu_{0}}\ell_{0}^{2}$ a dimensional constant ($\ell_{0}=7R_{E}$), *v* the solar wind bulk speed, *B* the interplanetary magnetic field magnitude, and $\theta_{c}$ the projection of the polar angle of the interplanetary magnetic field onto the *y*–*z* plane [11,26]

(2) $$\theta_{c}=\left\{\begin{array}{cc} \tan^{-1}\left(\left|\frac{B_{y}}{B_{z}}\right|\right), & \mathrm{if}\;B_{z}>0;\\ \pi -\tan^{-1}\left(\left|\frac{B_{y}}{B_{z}}\right|\right), & \mathrm{if}\;B_{z}<0;\\ \frac{\pi }{2}, & \mathrm{if}\;B_{z}=0. \end{array}\right.$$

We consider these two parameters, i.e., $B_{z}$ and ε, since they are directly connected to the energy, mass, and momentum transfers from the interplanetary space to the near-Earth electromagnetic environment (e.g., [1,10,11,26]). Indeed, when $B_{z}$ is southward oriented, solar wind particles are favourite to enter the magnetosphere via magnetic reconnection at the flanks of it, while when ε increases this means that a huge amount of energy is transferred to the magnetosphere.

Moreover, we also consider in our analysis geomagnetic indices data being representative of both magnetospheric and ionospheric current systems as the ring current and the auroral electrojects [27,28]. In particular we use the 5-minute time resolution low-latitude geomagnetic index SYM-H [27] which describes the geomagnetic disturbances at mid-latitudes in terms of symmetric (SYM) disturbances of the horizontal component (H) of the magnetic field near the equator, monitoring the variability of the magnetospheric ring current, and the auroral electroject (AE) indices, at 5 minutes resolution, obtained from high-latitude observations of the variability of the geomagnetic H component, monitoring the activity of the auroral electrojects [28].

As time interval for our study we collect a dataset consisting of the previous quantities/parameters over a 1-year period from 1 January 2000 to 1 January 2001, which corresponds to the maximum phase of solar cycle 23, such to have a considerable and sufficient statistics for correctly computing the information measures (e.g., the number of data points is N= 105,408).

**Figure 1:**
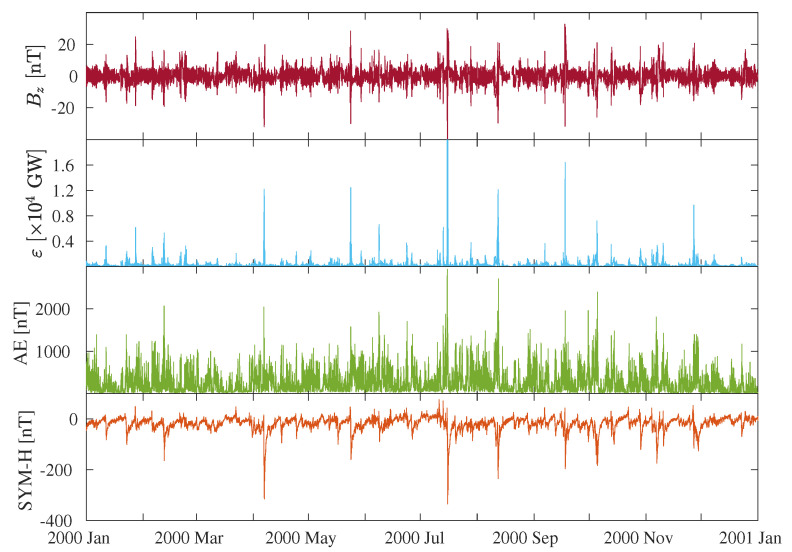
(From top to bottom) Solar wind parameters (magnetic field component $B_{z}$, ε parameter) and geomagnetic indices (AE and SYM-H), during the period 1 January 2000–1 January 2001, corresponding to the maximum phase of the 23th solar cycle.

The selected period (see Figure 1) is characterized by the occurrence of several geomagnetic storms and substorms, e.g., sudden increases of the intensity of the geomagnetic field due to enhancements in both magnetospheric and ionospheric current systems as a response to solar wind magnetic structures impacting the boundaries of the magnetosphere (e.g., [1,11]). One of these intervals corresponds to the well-known Bastille Day geomagnetic storm [12], e.g., the fifth strongest geomagnetic storm of the 23th solar cycle (Kp${}_{max}=9$, https://www.spaceweatherlive.com/en/auroral-activity/top-50-geomagnetic-storms/solar-cycle/23), associated with a fast moving Halo CME on 14 July 2000 at 10:54 UT, with a high transit time velocity (>750 km/s), and related to a forward interplanetary shock, observed at the L1 Lagrangian point on 15 July 2000 at 14:15 UT. This solar eruption was also accompanied by the emission of solar energetic particles observed at the Earth as an intense event [29]. During geomagnetic storms SYM-H tends to more negative values, meaning that the ring current is enhanced, and its evolution is firstly characterized by a rapid decreases (e.g., the storm main phase), followed by a very long and slow increase (e.g., the recovery phase) up to the unperturbed state (e.g., [12,27]). Conversely, geomagnetic substorms consist of explosive releases of stored magnetotail energy in the form of energetic particles and strong plasma flow. This results in an enhancement of auroral electrojets, followed by the excitation of discrete auroras becoming more intense and widespread, corresponding to larger values of the AE index (e.g., [11,28]). However, the great part of our dataset obviously consists of geomagnetically quiet periods, e.g., time intervals in which all currents residing in the near-Earth space are in their baseline levels (i.e., SYM-H ∼ 0 nT, AE < 100 nT).

## 3. Methods

Information theory methods are based upon the conceptualization of the information carried by a signal *X* in terms of the randomness associated to the probability distribution over the accessible states. In particular, the information content/randomness of a signal is provided by the *Shannon entropy* [30], defined as,

(3) $$H\left(X\right)=-\sum_{i=1}^{N}p\left(x_{i}\right)\log p(x_{i}),$$

where *x* is a variable which represents the possible outcomes of the process *X* and $p(x_{i})$ is the probability associated to the event $x_{i}$.

In the framework of information theory, more general and interesting functionals can be defined, as the Kullback-Leibler distance $D_{KL}$, i.e.,

(4) $$D_{KL}\left(p\left(x\right)||q\left(x\right)\right)=\sum_{i=1}^{N}p\left(x_{i}\right)\log\frac{p(x_{i})}{q(x_{i})},$$

where p(x) and q(x) are the observed distribution and a reference one for the outcomes of the process *X*, respectively, and the conditioned Kullback-Leibler distance $CD_{KL}$, i.e.,

(5) $$CD_{KL}\left(p\left(y|x\right)||q\left(y|x\right)\right)=\sum_{i=1}^{N}\sum_{j=1}^{M}p\left(x_{i},y_{j}\right)\log\frac{p(y_{j}|x_{i})}{q(y_{j}|x_{i})},$$

where p(y|x) and q(y|x) are the observed and reference conditioned distributions, respectively. These two functionals are usually named as *Kullback-Leibler divergences* (KLDs). It can be proven (e.g., [31]) that these measures satisfy the *Jensen’s inequality*, i.e., Equations (4) and (5) are always greater or equal to zero. In particular, the equality is satisfied if and only if *p* and *q* are equal, and the KLDs allow to define the distance from a suitable null hypothesis. For example, if we consider the hypothesis of statistical independence, e.g., being *X* and *Y* two random processes then p(x,y)=p(x)p(y), the so-called *mutual information*I(X,Y) can be derived from Equation (4) in the multivariate case as

(6) $$I\left(X,Y\right)=\sum_{i=1}^{N}\sum_{j=1}^{M}p\left(x_{i},y_{j}\right)\log\frac{p(x_{i},y_{j})}{p\left(x_{i}\right)p\left(y_{j}\right)},$$

providing I(X,Y) a general measure of correlation between two signals/processes since no hypothesis are made on the nature of both *X* and *Y* (e.g., [31]). More rigorously I(X,Y) quantifies the information shared between the two processes, i.e., the amount of inference on a process based on the knowledge of an other.

In real world physical systems, the maximal information shared between two processes, *X* and *Y*, could occur with a time delay τ; thus, it may be that I(X,Y)=0 in Equation (6) only because we are not considering the time delay between the two processes. In order to take into account the possible presence of a delay in the shared information, one can introduce a time delay τ in Equation (6), defining the so-called *delayed mutual information* (DMI), i.e.,

(7) $$DMI_{X,Y}\left(\tau \right)=I\left(X_{t},Y_{t+\tau }\right)$$

where τ is a time-lag. In such a way, using the DMI one can check for the occurrence of a certain shifting in time of correlation between *X* and *Y*, where the latter is shifted back in time. It is important to remark that the occurrence of a time lag in the maximal information shared between two processes has not to be confused as a measure of causality, that in such a framework should be identified only in the case of a directed flow of information. In other words, by only looking for correlation we are not able to conclude that $\tau^{*}$ is the causal time; indeed, the existence of a correlation time may be simply due to a common driver and only the past history of *X* does not improve the predictability of the τ-shifted *Y*.

In order to overcome this problem, Schreiber [32] introduced the concept of *transfer entropy* as the deviation from the generalized Markov condition

(8) $$p\left(y_{t+\tau }|y_{t}^{\left(k\right)},x_{t}^{\left(l\right)}\right)=p\left(y_{t+\tau }|y_{t}^{\left(k\right)}\right).$$

being $y_{t}^{\left(k\right)}=\left(y_{t},y_{t-1},...,y_{t-k+1}\right)$ and $x_{t}^{\left(l\right)}=\left(x_{t},x_{t-1},\ldots ,x_{t-l+1}\right)$. The *transfer entropy* $T_{X\to Y}\left(\tau \right)$ is based on transition probabilities, thus containing information about the dynamics underlying the processes *X* and *Y* [32], and can be obtained by inserting Equation (8) in Equation (5)

(9) $$T_{X\to Y}\left(\tau \right)=\sum_{y_{i+\tau },y_{i}^{\left(k\right)},x_{i}^{\left(l\right)}}p\left(y_{i+\tau },y_{i}^{\left(k\right)},x_{i}^{\left(l\right)}\right)\log\frac{p(y_{i+\tau }|y_{i}^{\left(k\right)},x_{i}^{\left(l\right)})}{p(y_{i+\tau }|y_{i}^{\left(k\right)})},$$

accounting for the information flow from *X* to *Y* [32]. This means that, if $T_{X\to Y}\neq 0$, the knowledge of the past history of *X* improve the prediction about the future of *Y*.

In the case of systems exhibiting dynamical complexity, the information exchange between variables is often bidirectional (feedback loop) [33]. For this reason, in our analyses we prefer to compute the *excess of transfer entropy* in terms of the difference between the transfer entropies from *X* to *Y* and *vice versa*, i.e.,

(10) $$\Delta T_{X↔Y}\left(\tau \right)=T_{X\to Y}\left(\tau \right)-T_{Y\to X}\left(\tau \right).$$

Thus, if $\Delta T_{X↔Y}\left(\tau \right)=0$ it means that the information flow from *X* to *Y* is balanced by the information flow from *Y* to *X*, so that Equation (10) is useful in order to unveil which variable, if there, dominates the interaction. This excess of transfer entropy in one specific direction represents a *net information flow* from one process to the other, which could support the possibility of a causality relation, i.e., a direct influence of a process on the other.

The numerical computation of the above quantities requires a certain attention. Indeed, in many situations when the state space of the processes under investigation is not extensively sampled, the application of histogram- and/or kernel-based methods for the evaluation of the joint distributions can produce fake results. To avoid as much as possible these computational problems, we follow the recipe described in reference [34] for the mutual information, which has been also implemented for the computation of the transfer entropy [35].

Clearly the algorithms are prone to the finite-size sample effects, as well as numerical ones (for instance, the transfer entropy will never be exactly equal to zero). For this reason, we need to estimate the bias by following a null-hypothesis test, thus for each computation of the net information flow we create the probability distribution of the values assuming the null hypothesis (absence of information flow) by means of surrogate time series [36]. The critical value to reject the null hypothesis is selected in correspondence of the value corresponding to the 95% of the probability distribution for the surrogates.

## 4. Results

**Figure 2:**
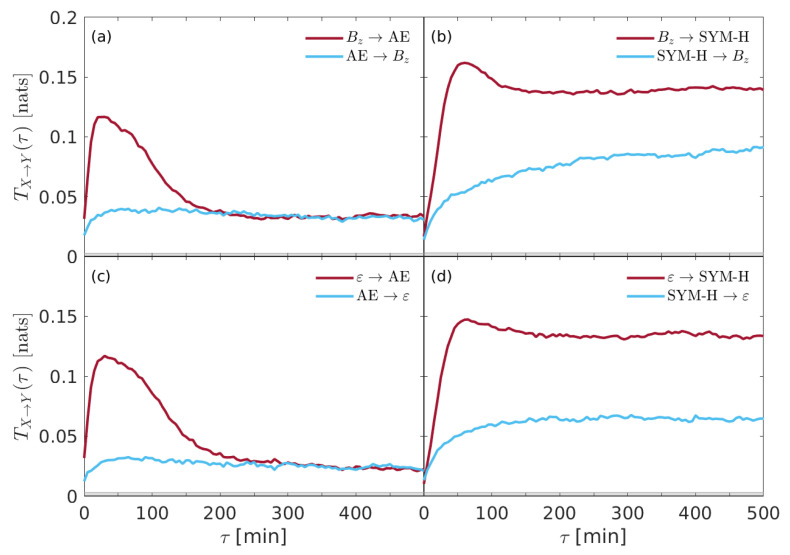
The transfer entropy $T_{X\to Y}\left(\tau \right)$ derived from each couple of time series and for both directions of the information flow. (**a**) $B_{z}⇌$ AE, (**b**) $B_{z}⇌$ SYM-H, (**c**) ε⇌ AE, and (**d**) ε⇌ SYM-H. The gray shaded area marks the 95% significance level.

We start our analysis on the information flow between solar wind parameters and geomagnetic indices by computing the direct and reverse flow of information between the different quantities. Figure 2 shows the transfer entropy $T_{X\to Y}\left(\tau \right)$ derived from each couple of time series and for both directions of the information flow (e.g., X⇌Y).

The results highlight that there is a strong, clear, and significant information transfer from both solar wind parameters (i.e., $B_{z}$ and ε) to both geomagnetic indices (i.e., SYM-H and AE). This is clearly an expected result, being the interplanetary medium variability the driver of the magnetosphere–ionosphere system dynamics. However, a different time delay τ is found for the two geomagnetic indices, while no significant variations are found for the two solar wind parameters. Indeed, a time delay τ∼30 min is found when solar wind information flows to the auroral ionosphere as monitored by AE-index, while τ∼60 min is found for the information transfer from solar wind to the inner and equatorial magnetospheric regions of the ring current as measured by SYM-H index. The first delay (τ∼30 min) is consistent with the response of the auroral electrojets, according to particles injection process, plus the time that the disturbance takes to reach the ionosphere from bow shock (e.g., [11,23]); the second time delay (τ∼60 min) is instead consistent with the characteristic times of convection processes (a slower phenomenon), plus the travel time of the disturbance to reach the inner magnetosphere from bow shock (e.g., [11,37]). The inverse directions, i.e., the information transfer from past values of geomagnetic indices to solar wind parameters, do not show any characteristic time delay. This could be interpreted as a fake result due to the inherent complex nature of the magnetospheric dynamics, which manifests in an unreasonable information transfer from the magnetosphere–ionosphere system to the near-Earth interplanetary medium. Forcing the interpretation of this reverse flow of information from the magnetosphere to the solar wind, the only possible conjecture that one could make is that the changes of the magnetospheric cavity and the nearby interplanetary medium could partially reflect in the interplanetary quantities. Clearly, this is a simple conjecture in an holistic point of view, which is very difficult to support on the basis of our simple analysis. Furthermore, larger values of the transfer entropy $T_{X\to Y}\left(\tau \right)$ are found when information flows from the solar wind parameters to SYM-H, while a lower information transfer is found for AE.

Whereas storms are surely correlated to the strong intensification of plasma flow (in terms of an intensification of the large plasma convection) inside the inner magnetosphere, substorms are phenomena, which are more related to the mechanisms through which the constantly flowing energy from the Sun is dissipated (e.g., [1,9,10,11]). When this coupling is strengthened by the arrival of a solar wind perturbation, both the intensity and the frequency of substorms, as monitored by auroral electrojet indices, grow up. Furthermore, it has been widely reported that the ion particle population of the ring current is due to both the solar wind and the terrestrial ionosphere. Indeed, although the quiescent ring current is mainly carried by protons of predominantly solar wind origin, during magnetic storms, the ionospheric plasma (mainly consisting of oxygen ions O${}^{+}$) is transferred to the magnetospheric regions via field aligned currents (FACs) [38]. This mechanism produces enhancements of the ring current intensity, especially during the main phase of a geomagnetic storm, also affecting the characteristic times of the recovery phase, e.g., the decay of the ring current, through charge exchange and wave-particle scattering loss mechanisms [39]. Thus, although geomagnetic storms are certainly driven by the solar wind variability and are associated with the enhancements of large-scale convective processes within the Earth’s magnetosphere, they could also be understood in terms of frequent and intense collection of substorms [40]. In this view, the coupling between geomagnetic storms and solar wind variability could be partially indirect, thus suggesting that the storm time ring current is increasingly terrestrial in origin [41]. This scenario is also supported by information theory-based results by De Michelis et al. [17], although recently Runge et al. [25], by applying a multivariate information-theoretic analysis, found no-evidence for a relation between high-latitude substorm phenomena and low-latitude storms.

**Figure 3:**
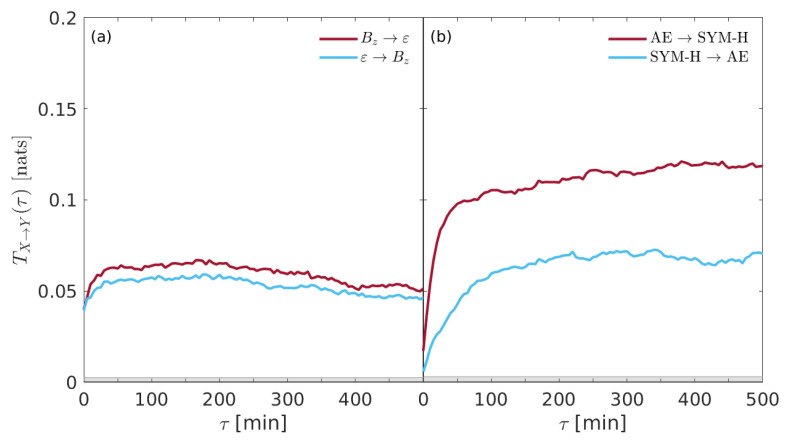
The transfer entropy $T_{X\to Y}\left(\tau \right)$ derived for (**a**) $B_{z}⇌\varepsilon$ and (**b**) AE ⇌ SYM-H, respectively. The gray shaded area marks the 95% significance level.

In order to deepen this subtle connection, it is helpful to investigate the information exchange between SYM-H and AE indices and also between $B_{z}$ and ε. Figure 3 reports the transfer entropy $T_{X\to Y}\left(\tau \right)$ derived from (a) $B_{z}⇌\varepsilon$ and (b) AE ⇌ SYM-H, respectively.

While it seems that no significant differences are found in the information flows between solar wind parameters, a clear difference emerges when geomagnetic indices (e.g., SYM-H and AE) are considered. Although no peaks are found for each transfer entropy, it seems that larger information is transferred from AE to SYM-H with respect to the inverse direction, e.g., from SYM-H to AE. This suggests that the internal dynamics of the near-Earth electromagnetic environment is characterized by an information exchange between the different regions.

To highlight which could be the driving variables in this complex system as well as to investigate the coupling strength between the different quantities, we evaluate the net information flow $\Delta T_{X↔Y}\left(\tau \right)$ as the difference of transfer entropies in both the direct and the inverse direction, e.g., $\Delta T_{X↔Y}\left(\tau \right)=T_{X\to Y}\left(\tau \right)-T_{Y\to X}\left(\tau \right)$. Here, $\Delta T_{X↔Y}\left(\tau \right)=0$ means the absence of information flow, while $\Delta T_{X↔Y}\left(\tau \right)≷0$ means that there exists an information flow from *X* to *Y* or vice versa.

**Figure 4:**
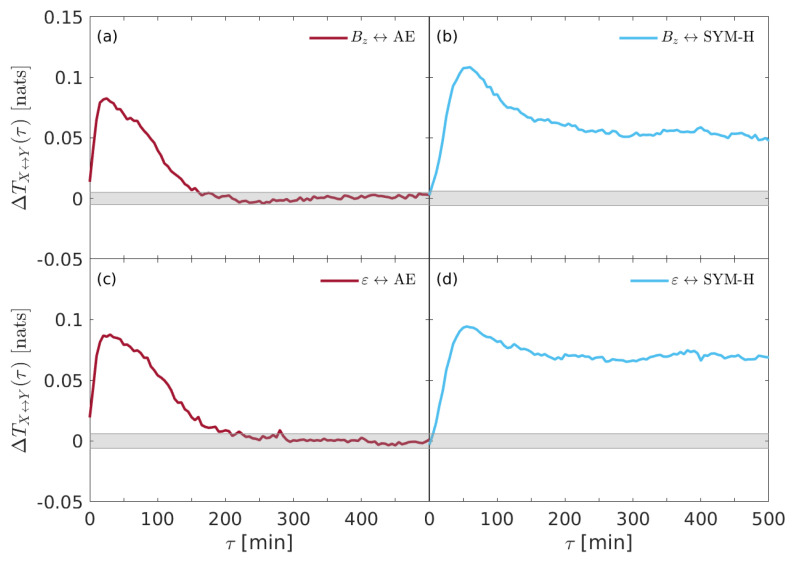
The net information transfer entropy $\Delta T_{X↔Y}\left(\tau \right)$ derived from the coupling between solar wind parameters and geomagnetic indices. (**a**) $B_{z}↔$ AE, (**b**) $B_{z}↔$ SYM-H, (**c**) ε↔ AE, and (**d**) ε↔ SYM-H. The gray shaded area marks the 95% significance level.

Figure 4 shows the net information flow $\Delta T_{X↔Y}\left(\tau \right)$ derived from the coupling between solar wind parameters and geomagnetic indices.

While ε and $B_{z}$ are equally significant for AE, it seems that $B_{z}$ is the most important variable for driving the behavior of SYM-H. Interestingly, AE seems to respond impulsively to external drivers; conversely, SYM-H is coupled for much more time, being the net information above the null hypothesis threshold for all the considered time delays.

Another interesting result seems to emerge when both the solar wind parameters and the geomagnetic indices are separately investigated by means of evaluating the net information flow between the two solar wind parameters (e.g., $B_{z}$ and ε) as well as between the two geomagnetic indice (e.g., SYM-H and AE).

**Figure 5:**
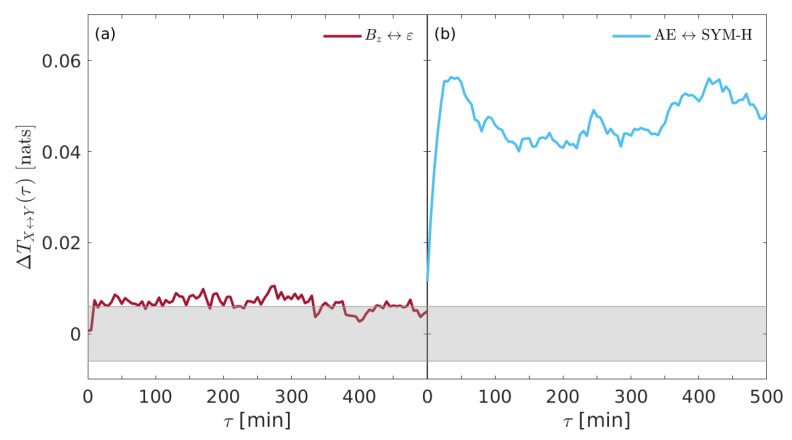
The net information transfer entropy $\Delta T_{X↔Y}\left(\tau \right)$ derived for (**a**) $B_{z}↔\varepsilon$ and (**b**) AE ↔ SYM-H. The gray shaded area marks the 95% significance level.

Figure 5 shows the net information flow $\Delta T_{X↔Y}\left(\tau \right)$ derived from the coupling between $B_{z}$ and ε (a) and between the two geomagnetic indices (b). It is clear that there is no a significant difference in the net information transfer between solar wind parameters, being $\Delta T_{B_{z}↔\varepsilon }\left(\tau \right)$ below the null hypothesis threshold for almost all time delays. Conversely, the information seems to be transferred predominantly from AE index to the ring current index (SYM-H), thus suggesting that substorms could drive the occurrence of storms to a certain extent. This result seems to support the previous findings by De Michelis et al. [17] and also the physical mechanisms about the ionospheric plasma outflow during geomagnetic storms suggested by Daglis et al. (e.g., [38,39,41]), although a different dataset (e.g., the whole year 2000 with respect to the whole year 1981) and a different proxy for the geomagnetic substorms (AE instead of AL) are considered. We will return on this point in the Section 5.

## 5. Discussion and Conclusions

In Section 4 we have presented the results on the information flow between interplanetary variables and geomagnetic indices which monitor the Earth’s magnetosphere–ionosphere system. One of the major outcome of our analysis is the signature of the primary role that the interplanetary magnetic field $B_{z}$ component plays in driving the Earth’s overall magnetospheric dynamics, while the Perrault-Akasofu ε coupling function seems to be less relevant than $B_{z}$ (e.g., Figure 2 and Figure 3). This may be interpreted as an evidence of the fact that the more relevant physical phenomenon responsible for plasma penetration within the magnetospheric boundaries is the magnetic reconnection, which principally occurs when the IMF is southward oriented. This result is extremely important in the framework of Space Weather forecast studies and support the feasibility to make predictive models exclusively based on the IMF field information content (e.g., [42]).

Moreover, it seems that the overall magnetospheric response to the solar wind variability occurs with a typical time delay of about 60 min, as monitored by the SYM-H index; conversely, the auroral activity typically enhances within 30 min. This difference can be interpreted in terms of the more complex dynamics of loading mechanisms of the ring current, which requires long-standing processes on longer timescales (e.g., [11] and references therein), as a stable convection allowing current intensification by plasma particle population. Indeed, the magnetospheric response is the result of the superposition of different processes occurring on a wide range of timescales: the shortest timescales, mainly related to the impulsive response, manifest into a strong information transfer (in the form of energy, mass, and momentum) in a short time, while the longer timescales, due to slow processes (convection, for example), although weaker, seem to dominate the overall information transfer with respect to the impulsive response. Conversely, interplanetary medium effects on high-latitude processes during geomagnetic substorms as monitored by AE-index seem to be characterized by an overall fast dynamics as justified by the fact that high latitudes are more subject to direct injection of particles from magnetotail.

Summarizing, our findings suggest that:

1. the solar wind drives both the magnetospheric and ionospheric variabilities as controlled by the SYM-H and AE indices, respectively. Thus, the solar wind-magnetosphere–ionosphere system is always connected with the information flow intensifying during strong interplanetary perturbations;
2. the most relevant variable, in terms of information flow toward SYM-H and AE, is $B_{z}$. On the average, by looking at the τ-shifted history of the interplanetary magnetic field carried by the solar wind one will be less uncertain about the future temporal variability of the SYM-H index when τ=60 min; on the other hand, to model AE index variability one has to look 30 minutes back in time at $B_{z}$;
3. the information flow from AE to SYM-H dominates with respect to the inverse direction. This means that, on the average, the auroral electrojets inject mass, energy and momentum into the ring current system in the magnetosphere, so that the information flow is not in a steady-state. This is an evidence of the fact that the ring current is enhanced by the energy flux coming from the ionospheric plasma outflows as already observed in reference [38].

**Figure 6:**
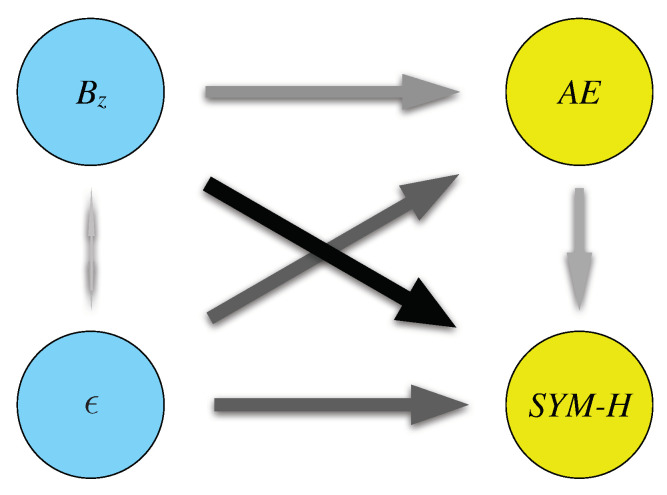
A sketch of the information flow among the different quantities here considered. The dimension of the arrows and their grey/black intensity indicates the relevance of the information flow.

These findings are schematised in Figure 6.

Comparing our results with previous studies (see, e.g., [17,25]) on a similar subject, we can note how our results support the recent results by Runge et al. [25] on the main role of $B_{z}$ in driving the magnetosphere–ionosphere response to the interplanetary medium variability. Conversely, regarding the information flow from high-latitude processes to low-latitude ones or vice-versa, our results seem to support the previous findings by De Michelis et al. [17] on the existence of an average driving of the ring current by high latitude phenomena. We remark that this possible link between geomagnetic substorms and storms is also supported by in-situ observations and other physical considerations [38,40]. The observed discrepancy with results reported in reference [25] could be due to the fact that here we are considering a different geomagnetic index, AE instead of AL, which is representative of the overall current flowing in the auroral ionosphere and not only of the westward electrojet. Moreover, another difference is the time resolution of geomagnetic indices: while we use 5-min resolution, Runge et al. [25] considered a smoothed version of geomagnetic indices at 20-min resolution. This obviously affects the monitoring of processes occurring on short timescales, being the auroral electrojects dynamics characterized by impulsive energy-release typically occurring on short timescales (e.g., [11,13,28,43]). Indeed, up to 60% of the AE-index bursts are characterized by a time duration of less than 20 min [13,43], so that the use of data with a temporal resolution of 20 min could not account for particle energisation that could contribute to the information flow towards the magnetospheric equatorial currents. This is a very relevant point that could explain the difference observed on the AE(AL)→SYM-H among this work, Ref. [17] and Runge et al. [25]. Clearly, this point deserves a more extended analysis on the timing of such an information flow that, however, at the moment is outside of the scope of this work.

In conclusion, here we have shown the suitability of information theory approaches to infer the nature of relationships and causal links in the study of the Earth’s magnetosphere–ionosphere system dynamics in response to interplanetary conditions changes. This approach is very promising in the field of Space Weather and will be pursued in forthcoming works by considering more events for enriching the statistics (e.g., by using an ensemble of geomagnetic storm and substorm periods) as well as by searching for a better understanding of the hypothesis of substorms-driving mechanism.

## Abbreviations

The following abbreviations are used in this manuscript:

AE	Auroral Electroject
CDAWeb	Coordinated Data Analysis Web
CME	Coronal Mass Ejection
DMI	Delayed mutual information
GSM	Geocentric Solar Magnetospheric System
H	Horizontal component
KLD	Kullback-Leibler Divergence
M-I	Magnetosphere-Ionosphere system
$R_{E}$	Earth radii
SPDF	Space Physics Data Facility
SW	Solar wind
SYM	Symmetric
